# Pandemic (H1N1) 2009 Outbreak at Canadian Forces Cadet Camp

**DOI:** 10.3201/eid1612.100451

**Published:** 2010-12

**Authors:** Rhonda Y. Kropp, Laura E. Bogaert, Robert Barber, Francois-William Tremblay, Robert Ennis, Martin Tepper, Robert Pless, Nathalie Bastien, Yan Li, Carole Beaudoin, James Anderson, Louise Pelletier, Rachel Rodin

**Affiliations:** Author affiliations: Public Health Agency of Canada, Ottawa, Ontario, Canada (R.Y. Kropp, F-W. Tremblay, R. Pless, L. Pelletier, R. Rodin);; Department of National Defence Headquarters, Ottawa (L.E. Bogaert, R. Barber, R. Ennis, M. Tepper, J. Anderson);; Public Health Agency of Canada, Winnipeg, Manitoba, Canada (N. Bastien, Y. Li, C. Beaudoin)

**Keywords:** Influenza A virus, subtype H1N1, disease outbreaks, viruses, young adult, military personnel, epidemiology, Canada, dispatch

## Abstract

We conducted a case–control study to describe the clinical and epidemiologic characteristics of an outbreak of pandemic (H1N1) 2009 at a Canadian military cadet training center. We found that asthma and obesity confer greater risk for infection. Viral shedding was detected by PCR up to 18 days after symptom onset.

On July 29, 2009, the Public Health Agency of Canada was notified of an outbreak of pandemic (H1N1) 2009 at the Army Cadet Summer Training Centre Argonaut at Canadian Forces Base, Gagetown, New Brunswick. The Cadet Summer Training Centre camp opened in early July and ran sessions lasting 2–6 weeks. The camp setting was semiclosed, with limited movement on and off camp. A case–control study was conducted to describe transmission, clinical characteristics, viral shedding, and risk factors for infection.

## The Study

Approximately 506 cadets, 12–18 years of age, and 322 staff cadets, officers, and support staff lived on camp premises. All persons at the camp were invited to participate. This study received expedited approval from the Health Canada Research Ethics Board. Participants were interviewed in person at the camp or by telephone; swab specimens were collected by on-site nurses. Samples were sent to the National Microbiology Laboratory for testing using reverse transcription–PCR and primer sets developed by the US Centers for Disease Control and Prevention (*1*). Specimens were cultured in primary CMK cells (Viromed Laboratories, Inc., Minnetonka, MN, USA) and the hemagglutinin titer was checked at days 6 and 10.

A modified case definition for pandemic (H1N1) 2009 infection was developed based on Canada’s surveillance case definition for influenza-like illness. Symptom onset was defined as earliest date of onset of self-reported history of fever or cough. The case definition is outlined in Table 1.

**Table 1:** Case definitions for pandemic (H1N1) 2009 infection, Army Cadet Summer Training Centre Argonaut at Canadian Forces Base, Gagetown, New Brunswick, Canada, 2009

Confirmed cases
Persons who
1. Had laboratory-confirmed pandemic (H1N1) 2009 influenza infection OR
2. Reported fever AND cough while at camp, excluding those who had negative PCR results for pandemic (H1N1) 2009 within 5 days after symptom onset
Suspected cases
Persons who
1. Reported fever OR cough with >2 of the following symptoms: sore throat, nausea, nasal congestion, chills OR
2. Reported fever and cough and had negative PCR results for pandemic (H1N1) 2009 within 5 days after symptom onset
Controls
Persons who
1. Did not report fever or cough OR
2. Reported fever or cough but without >2 of the following symptoms: sore throat, nausea, nasal congestion, chills

During August 3–27, 2009, we conducted 144 face-to-face and 21 phone interviews. Approximately 20% of cadets and 20% of staff cadets, officers, and support staff participated. Of the 165 participants, 56 were classified as confirmed cases, 24 as suspected cases, and 85 as controls. Participant age ranged from 13 to 43 years of age; 88% were 13–18 years, and 55% were male. No statistically significant demographic differences (p<0.05) were observed between confirmed cases, suspected cases, or controls.

The epidemic curve (Figure) summarizes the outbreak among those with known symptom onset date for cases (n = 54), suspected cases (n = 21), and 27 additional cases of fever and cough identified by the camp Health Care Centre (HCC) but not included in the study. The minimum camp attack rate for cases/suspected cases and HCC cases not in the study was 13.5% (112/828), 14.0% among cadets (71/506) and 12.7% among staff cadets and officers (41/322).

**Figure:**
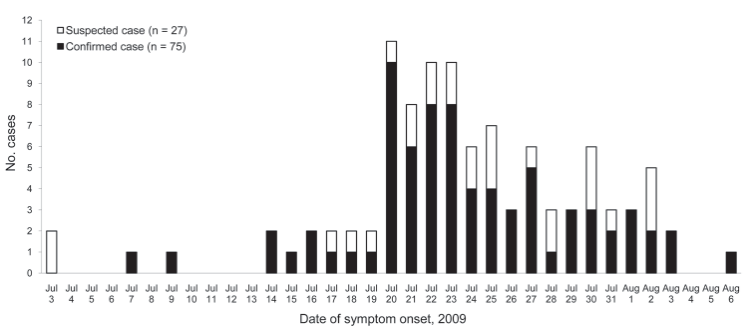
Epidemic curve of 54 confirmed and 21 suspected cases of pandemic (H1N1) 2009 infection and of 27 additional cases of fever and cough identified by the camp Health Care Centre, Army Cadet Summer Training Centre Argonaut at Canadian Forces Base, Gagetown, New Brunswick, Canada, 2009.

The outbreak was identified on July 17. In response, respiratory etiquette and hand hygiene were emphasized; camp residents were encouraged to seek care if ill. Those having fever and cough were isolated for 7 days, until parents came, or until laboratory results returned negative. Group outdoor activities stopped on July 23; all group activities were cancelled as of July 25. Some cadets were fast-tracked to graduate early, and arrival of new cadets was delayed 2 weeks. Mass screening for fever and cough was undertaken on August 6, before the arrival of new cadets; all new cadets were screened on arrival.

No activity or exposure was linked to increased risk for illness (data not shown). All but 1 person with a suspected or confirmed case reported symptoms; 58/85 (68.2%) of controls also reported symptoms during the outbreak period. Odds of experiencing shortness of breath, chest pain, sputum production, vomiting, rhinorrhea, nose bleeds, or change in level of awareness were all >5× higher for those with cases/suspected cases than for controls (Table 2). The mean number of symptoms among those with symptomatic cases/suspected cases was greater than among symptomatic controls (8.7 vs. 3.4; p<0.001). This relationship held true when comparing those with cases/suspected cases with symptomatic controls who had negative PCR results.

**Table 2:** Frequency of reported symptoms of pandemic (H1N1) 2009 infection, Army Cadet Summer Training Centre Argonaut at Canadian Forces Base, Gagetown, New Brunswick, Canada, 2009*

Symptoms	No. persons reporting symptom/no. persons reporting (%)			OR (95% CI)‡
	All participants, n = 165	Persons with confirmed/ suspected cases,† n = 80	Controls, n = 85	
None	28/165 (17.0)	1/80 (1.3)	27/85 (31.8)	
Systemic				
Fever	56/164 (34.1)	55/80 (68.8)	1/84 (1.2)	
Chills	44/165 (26.7)	41/80 (51.2)	3/85 (3.5)	
Headache	58/165 (35.2)	39/80 (48.8)	19/85 (22.4)	3.3 (1.7–6.5)§
Prostration	52/164 (31.7)	37/79 (46.8)	15/85 (17.6)	4.1 (2.0–8.4)¶
Malaise	67/165 (40.6)	47/80 (58.8)	20/85 (23.5)	4.6 (2.4–9.0)¶
Arthralgia	19/165 (11.5)	13/80 (16.2)	6/85 (7.1)	2.6 (0.9–7.1)
Myalgia	26/165 (15.8)	20/80 (25.0)	6/85 (7.1)	4.4 (1.7–11.6)§
Lower respiratory				
Cough	97/165 (58.8)	76/80 (95.0)	21/85 (24.7)	
Sputum production	33/164 (20.1)	26/79 (32.9)	7/85 (8.2)	5.5 (2.2–13.5)¶
Shortness of breath	30/165 (18.2)	26/80 (32.5)	4/85 (4.7)	9.8 (3.2–29.5)¶
Chest pain	14/165 (8.5)	13/80 (16.2)	1/85 (1.2)	16.3 (2.1–127.8)§
Upper respiratory				
Sore throat	76/164 (46.3)	56/80 (70.0)	20/84 (23.8)	
Nasal congestion	76/164 (46.3)	55/79 (69.6)	21/85 (24.7)	
Sneezing	27/164 (16.5)	20/79 (25.3)	7/85 (8.2)	3.8 (1.5–9.5)§
Runny nose	41/159 (25.8)	32/75 (42.7)	9/84 (10.7)	6.2 (2.7–14.2)¶
Nosebleeds	10/165 (6.1)	9/80 (11.2)	1/85 (1.2)	10.6 (1.3–86.1)#
Gastrointestinal				
Nausea	50/165 (30.3)	42/80 (52.5)	8/85 (9.4)	
Abdominal pain	28/165 (17.0)	17/80 (21.2)	11/85 (12.9)	1.8 (0.8–4.2)
Diarrhea	26/165 (15.8)	19/80 (23.8)	7/85 (8.2)	3.5 (1.4–8.8)#
Vomiting	24/165 (14.5)	20/80 (25.0)	4/85 (4.7)	6.8 (2.2–20.8)¶
Neurologic				
Seizures	0/164 (0.0)	0/80 (0.0)	0/85 (0.0)	NA
Change in awareness	29/165 (17.6)	25/80 (31.2)	4/85 (4.7)	9.2 (3.0–27.9)¶
Other				
Conjunctivitis	2/164 (1.2)	1/79 (1.3)	1/85 (1.2)	1.0 (0.1–17.5)
Other**	16/165 (9.7)	14/80 (17.5)	2/85 (2.4)	8.8 (2.0–40.1)§
Mean no. symptoms	6.5	8.7	3.4	

*One person reported no symptoms but was PCR and culture positive. OR, odds ratio; CI, confidence interval; NA, not applicable. †Fever and nausea reported by a greater proportion of cases than suspected cases (p<0.001 and p = 0.02, respectively). Cases and suspect cases did not differ significantly in frequency of symptoms not in the case definition or median number of symptoms and have therefore been combined here. ‡Odds ratios were not calculated for symptoms included in the case definition (fever, chills, cough, sore throat, congestion, nausea). §p<0.01. ¶p<0.001. #p<0.05. **Other symptoms reported: bilateral ear infection, bruising, ear pain, ear popping, eye pain, hot flashes, jaw pain, loss of appetite, loss of voice, rib pain, swelling of eyes, swollen tonsils, toothache (1 person each); dizziness (3 persons). All persons who reported symptoms in this category had >1 other reported symptom.

Of the 78 persons with symptomatic/suspected cases for whom complete information was available, 25 (32.1%) had recovered by the interview; median symptom duration was 7 and 9 days, respectively. Symptom duration >10 days was reported by 40% of persons with cases/suspected cases whose symptoms had resolved and 47% of those with unresolved symptoms. Median time from symptom onset to illness peak was 2 days (range 1–14 days). With the exception of cough, sputum production, and malaise, symptoms peaked rapidly (24–48 hours) after onset.

Overall, 86.1% of persons with cases/suspected cases accessed the HCC; none were hospitalized. Oseltamivir was given to 2 persons with confirmed cases who had comorbid conditions (asthma and kidney disease). Forty-four persons with cases/suspected cases (55.7%) were not isolated because they did not seek treatment at the HCC or not while both fever and cough were present.

Eight persons had positive PCR results for pandemic (H1N1) 2009 7–18 days after symptom onset, and live virus was detected up to 14 days after symptom onset. All but 1 of these persons were capable of transmitting virus given upper respiratory symptoms, and 2 reported diarrhea and vomiting on the day the swab sample was obtained. Four persons had live virus detected after day 7 of illness (up to 14 days); 2 of these reported comorbid conditions.

Persons with confirmed and suspected cases did not differ with regard to comorbidity or risk factors, except for seasonal influenza vaccination; 6/48 (12.5%) of persons with confirmed cases reported having received the seasonal influenza vaccine in the year of the study versus 8/22 (36.4%) of those with suspected cases (odds ratio 4.0; p<0.05). No difference was found in the proportion of those with cases/suspected cases and controls reporting seasonal influenza vaccination during the current year or past 2 years.

The odds of reporting >1 comorbidity was >2.7× higher for persons with cases/suspected cases than for controls (p<0.05) and for asthma >3.9× higher (p < 0.05). The odds of being obese were >3× higher for persons with case/suspected cases (odds ratio 3.4, 95% confidence interval 1.0–10.9).

## Conclusions

In accordance with national recommendations (*2*), antiviral drugs were not used for control; transmission appeared to be reduced through nonpharmaceutical measures. Multiple index cases could not be ruled out. No individual activity or exposure was linked to increased risk for illness.

High rates of obesity have been noted among hospitalized patients with pandemic (H1N1) 2009 (*3**–**6*). This study suggests obesity is a risk factor for infection or clinical illness and given low prevalence of comorbid conditions may stand alone as a risk factor. Consistent with international studies (*7*), vaccination for seasonal influenza was neither protective nor a risk factor for acquiring pandemic (H1N1) 2009. One third of case-patients reported change in level of awareness, which suggests the potential for mild neurologic sequelae. Neurologic complications of influenza infection have been reported in hospitalized children (*8**,**9*).

Seven of 8 participants who had positive PCR results for pandemic (H1N1) 2009 >7 days after symptom onset were capable of transmitting virus, given their upper respiratory symptoms on the day the swab sample was obtained. Studies of seasonal influenza indicate median viral shedding of 7–8 days after illness onset, with titers low or undetectable by day 5, although prolonged shedding has been reported (*10**–**13*). In this study, test results for pandemic (H1N1) 2009 were positive by culture up to 14 days and by PCR up to 18 days after symptom onset. This and other studies describe longer shedding periods for pandemic (H1N1) 2009 as compared with seasonal influenza (*10**,**13**–**15*).

Study limitations should be acknowledged. The case definition included self-reported fever and cough. Therefore, misclassification of persons with illnesses other than pandemic (H1N1) 2009 may have occurred. Convenience sampling was used so participants may differ from nonparticipants; complete demographics for the camp were not available for comparison. A small proportion of phone interviews were conducted <6 weeks after symptom onset, raising the possibility of recall bias.

Infection control procedures likely contributed to the control of transmission in the absence of antiviral drug use or early treatment for contacts. Shedding in otherwise healthy adolescents and young adults may be longer than shedding of seasonal influenza viruses, which may have implications for public health planning.
